# Things Practical in Dental Practice

**Published:** 1893-08

**Authors:** J. G. Templeton

**Affiliations:** Pittsburg, Pa.


ARTICLE III.

### THINGS PRACTICAL IN DENTAL PRACTICE.

J. G. TEMPLETON, D. D. S., PITTSBURG, PA.

At the Ohio State Dental Society, Dr. Templeton gave an interesting talk on the above subject. Some of the ideas given were as follows :

INSTRUMENT POLISHER.—Burnishers give better results when new than when tarnished, and it is essential to keep them finely polished. In fact, it is desirable to keep all instruments polished. An efficient device for polishing can be made by fastening a piece of sole leather, or a piece of razor-strop, on a block of wood of suitable size, and placing a little diamantin powder on the surface of the leather; then polish instruments by rubbing briskly on this surface. Diamantin is used by jewelers, and can be obtained from them or from their supply houses.

TO MAKE MOISTURE TIGHT GUTTA-PERCHA FILLINGS.—Take common resin and dissolve in chloroform to desired thickness; place some of this in the prepared cavity, and by the time the gutta-percha is heated, the varnish will be in proper condition through evaporation of the chloroform. The varnish should not extend to the cavity margins. Apply the gutta percha as usual, and pack with cold instruments. The cold instruments do not adhere as warm ones do. When completed the filling may be pared off to the proper contour by means of a heated thin blade instrument, and the filling smoothed by the application of eucalyptol or oil of cajuput.

TO DUPLICATE MODELS AND IMPRESSIONS.—Take printers' roller composition, melt in a water-bath till dissolved. Grease the model slightly with lard, and place it the same as if to mold a metal die, cover with a metal ring (a tin can opened at both ends will do), and pour the melted composition over the model. Let this stand over night. By morning the material is hardened and the model can be withdrawn. The composition being elastic it retains its shape. and a hundred models may be poured if necessary. Impressions may be duplicated in the same manner, by using impressions instead of model.

A USEFUL CLAMP.—Where the lower teeth have short and tapering crowns and it is impossible to make an ordinary clamp hold, use the Lyder clamp, and you will be successful.

TO DRY A CAVITY BEFORE FILLING.—After applying absolute alcohol to the cavity, use a solution of sandarach and ether to line the cavity; dry this with hot air, which forces it into the ends of the tubules, completely sealing them; then proceed with the filling.

IN LIGATING RUBBER-DAM, tie a small bead on the ligature, which, when tied around the tooth, will prevent the dam from coming over ligature; the bead should be on lingual side of tooth.

IN ARTICULATING TEETH, always take an impression of lower teeth when making an upper set. and in taking the bite have wax trimmed to show the length you wish the teeth to be, and bite into it just sufficiently to show the tips of cutting edges and cusps where the model made from lower impression can be placed in proper position, etc. For double sets, make wax models for contour in restoration of features and to show length of teeth, and then try these models in the mouth, being careful to see that you have it right; then make plaster articulating models for setting up the teeth, setting up the lower ones first against a plaster articulating plate, its articulating surface corresponding with the articulating surface of lower wax model, then lay aside the plaster articulating plate and put the model of upper jaw in its place, and set the upper teeth to the lower ones. I adopted this method about twenty-four years ago, and in that length of time have not had to grind a cusp off to let front teeth come together, and can say the same for the method of making an upper set alone, which is all due to the care taken to get a correct bite in such cases by taking an impression of lower teeth, which takes a little more time, but is all remunerated for in the satisfaction one gets from seeing there is nothing more to do when the piece is placed in the mouth with masticating surfaces perfect, and no need of any "grinding in" to get the front teeth together.

TO PREVENT PLASTER ADHERING TO RUBBER PLATES. —Coat the model with a thin solution of soap and water just before packing the case.

A METHOD OF SECURING PERFECT IMPRESSIONS FOR PARTIAL UPPER PLATES.—To take an accurate impression of the mouth for a partial upper set of teeth, smear plaster over the roof of the mouth with the finger, take a string about one foot in length, tie the ends together, put the tied end of the loop into the plaster on the roof of the mouth, and add more plaster to thoroughly imbed the knot, leaving the loop of string hanging down. In placing the plaster in the mouth care should be taken to have it come full half way over the grinding surfaces of molars and bicuspids and cutting edges of the front teeth, then trim the plaster and varnish the trimmed surfaces. The plaster should be so trimmed that it will fill up fully one-half of all spaces between the teeth, then cover all the remaining surface of the mouth and teeth with plaster, being very careful to have the teeth well covered and spaces filled in putting on plaster for the buccal and labial surfaces. When set, the plaster impression readily parts where it has been varnished, the palatial portion is dislodged with the help of the string used, and the pieces are then placed together and model made. If a tooth is irregular, use modeling compound about it and trim suitably; then apply the plaster. If in removing it breaks where joined, remove the compound, place in position in the impression and pour the model.—*Office and Laboratory.*

